# Caffeine supplement, inflammation, and hepatic function in cirrhotic patients: A randomized, placebo- controlled, clinical trial

**DOI:** 10.1016/j.heliyon.2024.e41138

**Published:** 2024-12-11

**Authors:** Seyed Ali Abbas-Hashemi, Zahra Yari, Behzad Hatami, Amir Anushiravani, Shadi Kolahdoozan, Ali Zamanian, Nadia Akbarzadeh, Azita Hekmatdoost

**Affiliations:** a Department of Clinical Nutrition and Dietetics, Faculty of Nutrition Sciences and Food Technology, National Nutrition and Food Technology Research Institute, Shahid Beheshti University of Medical Science, Tehran, Iran; b Department of Nutrition Research, National Nutrition and Food Technology Research Institute and Faculty of Nutrition Sciences and Food Technology, Shahid Beheshti University of Medical Sciences, Tehran, Iran; c Research Institute for Gastroenterology and Liver Diseases, Shahid Beheshti University of Medical Science, Tehran, Iran; d Digestive Diseases Research Center, Digestive Diseases Research Institute, Tehran University of Medical Sciences, Tehran, Iran

**Keywords:** Caffeine, Cirrhosis, Inflammation, Fibrosis

## Abstract

**Aim:**

We investigated the possibility of caffeine supplementation for managing the inflammation, and hepatic function in cirrhotic patients.

**Methods:**

In this randomized, double-blind, placebo-controlled trial, fifty patients with cirrhosis were randomly assigned to receive either caffeine supplement (400 mg), or placebo for eight weeks.

**Results:**

The results indicated a significant decrease in AST, platelets (P = 0.002), and PTT (P < 0.001), in the caffeine group compared to the placebo group. Also, caffeine supplementation resulted in a significant reduction in inflammatory biomarkers compared to placebo (p < 0.05). A significant improvement in liver indices including AST to platelet ratio index (APRI), (P < 0.001). Fibrosis 4 score (P < 0.001), and MELD score (P = 0.034)., was observed after 8 weeks caffeine supplementation.

**Conclusion:**

The results of the present study indicated that daily supplementation of 400 mg caffeine in cirrhotic patients can significantly improve liver fibrosis and reduce inflammatory factors.

The trial was registered at the Iranian Registry of Clinical Trials (Registration ID: IRCT20100524004010N34).

## Highlights

• Caffeine presents anti-inflammatory and anti-fibrosis effects, which can play an important role in the treatment, and prevention of complications and mortality of hepatic cirrhosis.
• Caffeine supplementation in dose of 400 mg/day improves hepatic functions in patients with cirrhosis.
• Consumption of 400 mg/day Caffeine can reduce inflammation in cirrhotic patients.

## 1 Introduction

Cirrhosis is the final stage of liver disease with necrosis and fibrosis of hepatocytes [1]. Cirrhosis is considered a major health problem with annual exceeding morbidity and mortality rate [2], while about one million deaths per year are attributed to this disease [3]. The one-year mortality of cirrhosis is estimated between 1 and 57 percent depending on the stage of the disease [4]. The prevalence of cirrhosis is increasing and merely one out of three people who are suffering from this disease, are aware of their disease. The most important causes of cirrhosis include chronic alcoholic fatty liver disease, viral hepatitis, and non-alcoholic fatty liver disease (NAFLD) [2].

Accumulating evidence suggests that dietary factors including antioxidants play critical role in the cirrhosis management. Dietary intervention in hepatic cirrhosis aims at minimizing and correcting malnutrition, preventing the progression of liver failure, and managing liver cirrhosis complications. The continuous inflammatory stimuli and oxidative stress can increase the risk of fibrosis and cancer [5]. It has been shown that there is a reverse association between total dietary antioxidant capacity and liver damage in patients with non-alcoholic steatohepatitis [6]. Although the role of nutrition has been well-documented in the prevention and treatment of cirrhosis, few studies have investigated dietary components that may exert protective effects on cirrhosis [7,8].

Coffee constitutes a high percentage of total dietary antioxidants[9,10], which is mainly attributed to its caffeine content [11]. The inverse relationship between coffee consumption and serum levels of alanine aminotransferase (ALT) and gamma-glutamyl transferase (GGT) has been demonstrated, which are markers of liver damage and indicator of liver fibrosis [[12], [13], [14]]. Moreover, it has been shown that coffee can prevent the development of hepatocellular carcinoma caused by liver fibrosis and cirrhosis [15,16]. In addition, the increase in coffee consumption has been associated with a decrease in the spread of liver fibrosis [17], which can be attributed to its caffeine content. Some of the effects of caffeine include inhibition of transcription factor Snail-1, down-regulation of profibrogenic genes, and activation of Nrf2, which induce the system antioxidant enzymes and thus prevent inflammation and fibrosis [18]. Although the relationship between coffee consumption and reduction of the risk of liver diseases, including cirrhosis, has been previously reported in epidemiological studies, the results of these studies have been contradictory [[19], [20], [21]].

Considering the anti-inflammatory and anti-fibrosis effects of caffeine, it seems that it can play an important role in the treatment, prevention of complication and mortality of hepatic cirrhosis. To the best of our knowledge, no clinical trial study has been published on the effects of caffeine supplementation in cirrhotic patients. The present study aimed to evaluate the effects of caffeine supplementation on hepatic outcomes, disease severity scores and inflammatory biomarkers in cirrhotic patients.

## 2 Materials and methods

### 2.1 Participants

This parallel, randomized, double-blind, placebo-controlled clinical trial, performed for eight weeks in patients with hepatic cirrhosis. Patients were recruited from Taleghani Hospital and Shariati Hospital, Tehran, Iran from June 2023 to September 2023. Before the onset of the trial, the sample size was calculated according to an investigation which was performed by Nouri-Vaskeh et al. [22], with a statistical power of at least 80 %. In this study, the number of samples in each group was estimated to be 22 patients. Considering a drop-out rate of 10 %, 25 patients were enrolled in each group.

Cirrhosis was diagnosed through biopsy or clinical assessment, including liver stiffness >12.5 kPa according to transient elastography. Patients diagnosed with hepatic decompensated cirrhosis, aged 18–75 years, with BMI of 18.5–30 kg/m^2^ were included. To minimize the covariates effects, we included only cirrhotic patients induced by viral hepatitis, fatty liver disease, and autoimmune hepatitis. Patients with a history of extrahepatic organ failure, taking caffeine-containing drugs or supplements, consuming more than 3 cups of coffee per day, consuming alcohol, and pregnant or breastfeeding mothers were abandoned from the study. The exclusion criteria were unwilling to continue cooperation, consumption of less than 80 % of the supplements, lost more than 10 % of body weight during the study, and getting pregnant.

### 2.2 Study design

To randomly assign patients to caffeine or placebo group, blocked randomization method was used and patients were stratified based on body mass index (BMI) as >25, and equal or more than 25. Participants in the caffeine group received 400 mg of caffeine per day (two 200 mg caffeine tablets, Raha Co, Iran) for 8 weeks. While, the placebo group received two pills containing starch powder with the identical shape, color, and size as caffeine supplements (Raha Co, Iran). The participants were advised to take the tablets twice per day with the breakfast and dinner. None of the participants and researchers were aware of the contents of the pills and the randomization procedure. Patients were also asked not to change their diet and physical activity. At each visit, information was gathered regarding adverse events and adherence to study. The compliance of the participants was evaluated by weekly phone calls and counting the remaining pills in containers at each follow-up visits.

### 2.3 Ethics and informed consent

Written informed consent to publish the clinical data was acquired from each participant prior the enrolment. This study was approved by the ethics committee of the Shahid Beheshti University of Medical Sciences on 22/May/2022 (Ethical ID: IR. SBMU.NNFTRI.REC.1401.009), and the trial was registered at the Iranian Registry of Clinical Trials (Registration ID: IRCT20100524004010N34).

### 2.4 Assessment of biochemical indicators

After 12-h overnight fasting 10 ml venous blood samples were taken by a trained nurse at the baseline and at the end of the intervention. Blood samples were collected in EDTA purple top tubes, and plain red top tubes for hematologic, and biochemical assessments, respectively. The plain tubes were centrifuged instantly, and serum were separated and stored at −80° centigrade until analysis. Serum concentrations of creatinine, bilirubin total, bilirubin direct, aspartate aminotransferase (AST), ALT, and alkaline phosphatase (ALP) were determined enzymatically (Delta Darman, Tehran, Iran). Albumin was assessed by the photometric method (Delta Darman, Tehran, Iran). Also, Serum concentrations of sodium and potassium were measured by the Ion Selective Electrode method. To assess Interleukin-6 (IL-6), the Electrochemiluminescence method (Roche, Indianapolis, USA) was used. High-sensitivity C-reactive protein (hs-CRP) was measured by immunoturbidimetry (Pars Azmoon, Tehran, Iran) and serum levels of tumor necrosis factor alpha (TNF-α) were quantified, using ELISA method. Prothrombin time (PT) and partial thromboplastin time (PTT) were quantified using a coagulation method by coagulometer. Platelet (PLT) was assessed using the counting blood cell method by Sysmex KX-21N Hematology Analyzer.

### 2.5 Anthropometric assessment

Basic and demographic characteristics of participants were collected through general questionnaire. Weight was measured without shoes and minimal clothes by a manual scale to the nearest 100 g. Height was evaluated in a standing position without shoes by a tape meter to the nearest 0.5 cm. We calculate BMI by dividing the weight in kilograms by the square of the height in meters.

### 2.6 Dietary assessment

Three 24-h dietary recall were recorded of each patient at the first and end of the study, including two weekdays and one weekend. The first recall was completed through face-to-face interview and two others were recorded by phone interviews. Dietary assessment was performed by a skilled nutritionist.

### 2.7 Statistical analysis

Statistical analysis was conducted by SPSS version 24 (SPSS Inc, Chicago, IL, USA). Descriptive statistics were used to illustrate the characteristics of the participants. We used the chi-square test to ascertain the differences between the qualitative variables of the two groups. An Independent sample *t*-test was performed to determine the differences in hepatic and inflammatory index between the two groups, and for evaluating the difference between the mentioned variables within the groups we used paired sample *t*-test. The data are illustrated as mean ± standard deviation for continuous variables and number (percentages) for categorical variables. We performed the analysis of covariance test to exclude the effects of confounding factors. We adjusted all analysis of covariance (ANCOVA) tests for the baseline value of each variable. P value less than 0.05 is considered significant.

## 3 Results

**Fig. 1:**
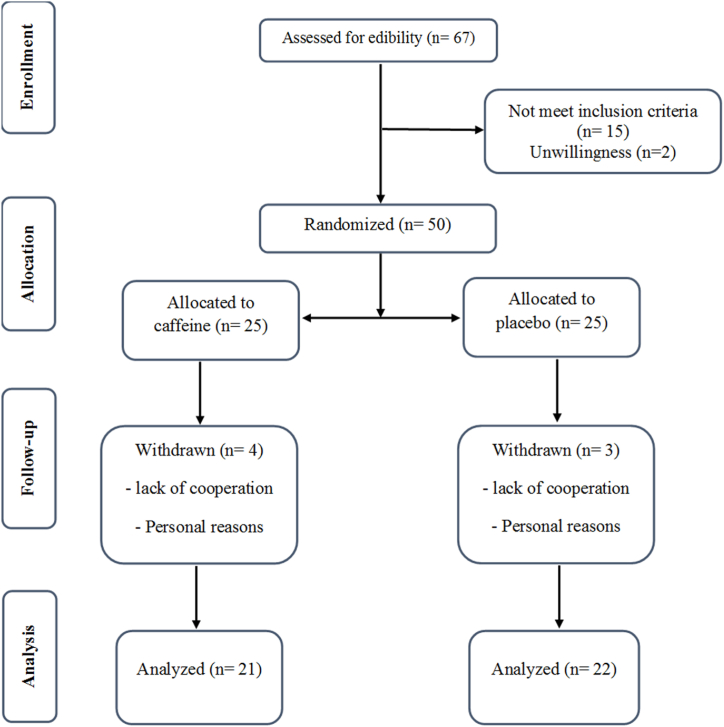
Flow diagram of participants recruitment.

Fifty patients have been enrolled in the present study. The flow chart of study enrollment is presented in Fig. 1. Forty-three patients completed 8 weeks of this study. Four patients in the intervention group and three patients in the placebo group discontinued the trial because of personal reasons and lack of cooperation. The compliance of participants was over 85 %. In this study, no adverse events were reported.

The baseline characteristics of the patients did not differ significantly between the two groups (Table 1). As shown in Table 2, there was no significant difference in the serum concentration of biochemical parameters, except PT, between the two groups. The results of the paired *t*-test indicated significant alterations in liver enzymes (AST and ALT) and coagulation factors (platelet, PT, PTT and international normalized ratio (INR)) in the caffeine group during the intervention, while in the placebo only platelet showed a significant reduction. Based on ANCOVA analysis, caffeine supplementation compared to the placebo group was able to significantly change AST (−6.7 ± 7.44 vs −1.14 ± 9.4, P = 0.014), platelet (12.2 ± 18.12 vs −18.4 ± 36.99, P = 0.002), and PTT (−2.72 ± 5.5 vs −0.14 ± 4.5, P < 0.001).

**Table 1:** Baseline characteristics of cirrhotic patients in the caffeine group and the placebo group.

	Caffeine	Placebo	*p*-value
**Sex (M/F)**	5/15	11/11	0.121
**Smoker**	7	5	0.499
**Age (y)**	53.4 ± 8.3	50.2 ± 13.6	0.373
**Weight (kg)**	73.5 ± 14.7	74.9 ± 15.4	0.761
**Height (cm)**	167.7 ± 8.2	166.1 ± 9.3	0.562
**BMI (kg/m**^**2**^**)**	26.1 ± 4.6	27.1 ± 4.8	0.498
**Etiology of Cirrhosis**			
**Viral Hepatitis**	8	9	1
**Fatty Liver Disease**	8	9	
**Autoimmune Hepatitis**	4	4	

Values are means ± SDs for continuous variables and number for categorical variables.

ANOVA for quantitative variables and χ2 test for qualitative variables.

BMI body mass index.

**Table 2:** The mean and standard deviation of the concentration of serum parameters and its changes in cirrhotic patients in the caffeine group and the placebo group.

	Baseline	After 8 weeks	Within-group *P* value	Changes	*P* value^b^
**Total Bilirubin**					0.385
Caffeine	1.11 ± 1.05	1.02 ± 0.74		−0.09 ± 0.56	
Placebo	1.14 ± 0.63	1.15 ± 0.5	0.482	0.007 ± 0.51	
*P* value^a^	0.923	0.532	0.947	0.558	
**Albumin**					0.174
Caffeine	3.57 ± 0.43	3.49 ± 0.47	0.440	−0.078 ± 0.44	
Placebo	3.66 ± 0.60	3.73 ± 0.60	0.453	0.067 ± 0.41	
P value	0.590	0.167		0.277	
**AST**					0.014
Caffeine	38.1 ± 17.75	31.4 ± 15.91	0.001	−6.7 ± 7.44	
Placebo	39.64 ± 16.45	38.18 ± 12.33	0.476	−1.14 ± 9.4	
P value	0.733	0.129		0.054	
**ALT**					0.448
Caffeine	30.25 ± 13.81	26.8 ± 13.5	0.001	−3.45 ± 3.99	
Placebo	34.64 ± 17.42	32.05 ± 15.04	0.320	−2.59 ± 11.93	
P value	0.375	0.243		0.75	
**ALP**					0.497
Caffeine	203.75 ± 56.95	197.9 ± 64.26	0.629	−5.58 ± 53.21	
Placebo	206.3 ± 112.5	211.14 ± 96.47	0.733	4.77 ± 64.83	
P value	0.926	0.608		0.567	
**Platelet**					0.002
Caffeine	120.84 ± 73.2	133.05 ± 71.13	0.009	12.2 ± 18.12	
Placebo	126.95 ± 59.7	105.55 ± 50.7	0.030	−18.4 ± 36.99	
P value	0.655	0.207		0.002	
**Creatinine**					0.130
Caffeine	1.03 ± 0.5	0.91 ± 0.31	0.081	−0.12 ± 0.28	
Placebo	0.8 ± 0.21	0.87 ± 0.22	0.103	0.06 ± 0.18	
P value	0.063	0.587		0.016	
**Serum Na**					0.113
Caffeine	147.75 ± 6.95	150.95 ± 11.08	0.267	3.2 ± 12.5	
Placebo	145.63 ± 12.28	138.26 ± 29.9	0.213	−7.36 ± 26.9	
P value	0.501	0.082	0.117	0.117	
**INR**					0.458
Caffeine	1.07 ± 0.14	1.17 ± 0.15	0.025	0.09 ± 0.17	
Placebo	1.14 ± 0.12	1.16 ± 0.13	0.578	0.017 ± 0.14	
P value	0.197	0.867		0.117	
**PT**					0.757
Caffeine	12.92 ± 1.06	13.77 ± 1.16	0.018	0.84 ± 1.41	
Placebo	14.2 ± 1.73	14.25 ± 1.6	0.863	0.05 ± 1.46	
P value	0.013	0.281		0.088	
**PTT**					0.000
Caffeine	34.2 ± 4.66	31.47 ± 2.38	0.047	−2.72 ± 5.5	
Placebo	35.84 ± 4.15	35.7 ± 3.53	0.882	−0.14 ± 4.5	
P value	0.309	0.000		0.111	

Values are means ± SDs.

AST: aspartate transaminase; ALT: alanine transaminase; ALP: alkaline phosphatase; INR: international normalized ratio; PT: prothrombin time; PTT: partial thromboplastin time.

a Between-group P value: independent *t*-test.

b Analysis of covariance (ANCOVA) tests for the baseline value of each variable.

As shown in Table 3, at the beginning and end of the study, there were no significant differences between the two groups in terms of inflammatory biomarkers. All three inflammatory biomarkers (hs-CRP, IL-6 and TNF-a) in the caffeine group showed a significant decrease during the study, while in the placebo group only TNF-a increased significantly (P = 0.02). After adjusting the baseline value of each variable, the analysis of the ANCOVA showed that caffeine supplementation can reduce inflammatory factors significantly (Table 3).

**Table 3:** The mean and standard deviation of the inflammatory factors in the caffeine group and the placebo group.

	Baseline	Week 8	Within-group *P* value	*P* value^b^
**hs-CRP**				0.007
Caffeine	2.17 ± 1.98	1.46 ± 1.42	0.017	
Placebo	2.1 ± 1.47	2.38 ± 1.56	0.084	
*P* value^a^	0.699	0.148		
**IL-6**				0.014
Caffeine	4.59 ± 5.55	3.44 ± 4.5	0.001	
Placebo	5.03 ± 4.01	5.22 ± 3.63	0.404	
*P* value	0.835	0.307		
**TNF-α**				0.000
Caffeine	9.15 ± 7.5	6.5 ± 6	0.002	
Placebo	5.76 ± 2.5	6.59 ± 2.35	0.02	
*P* value	0.085	0.967		

Values are means ± SDs.

hs-CRP: high sensitivity C-reactive protein; IL-6: interleukin 6; TNF- α: tumor necrosis factor alpha.

a Between-group P value: independent *t*-test.

b Analysis of covariance (ANCOVA) tests for the baseline value of each variable.

**Fig. 2:**
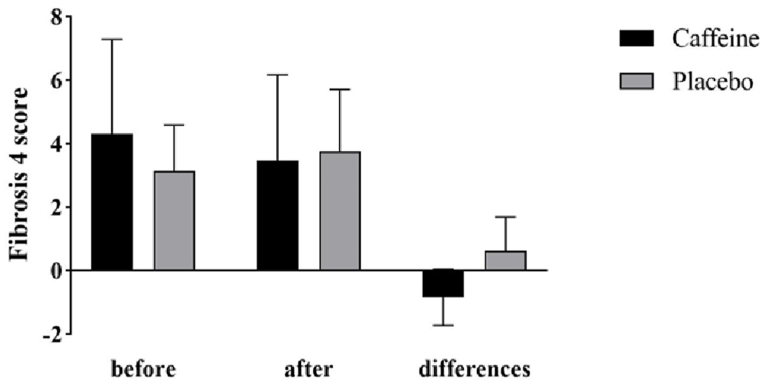
Fibrosis 4 score and its changes in cirrhotic patients in the caffeine group and the placebo group.

**Fig. 3:**
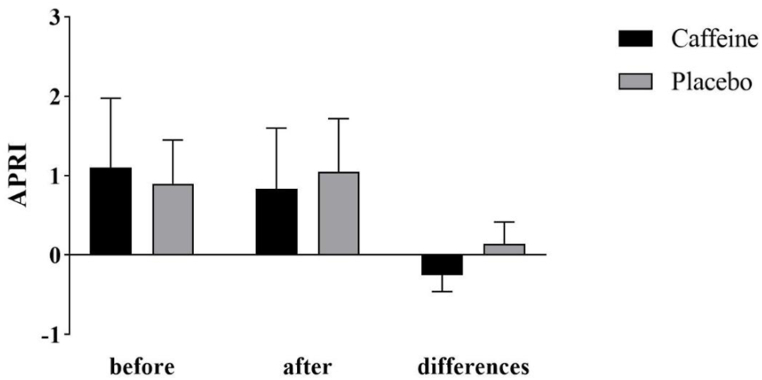
AST to Platelet Ratio Index (APRI) and its changes in cirrhotic patients in the caffeine group and the placebo group.

**Fig. 4:**
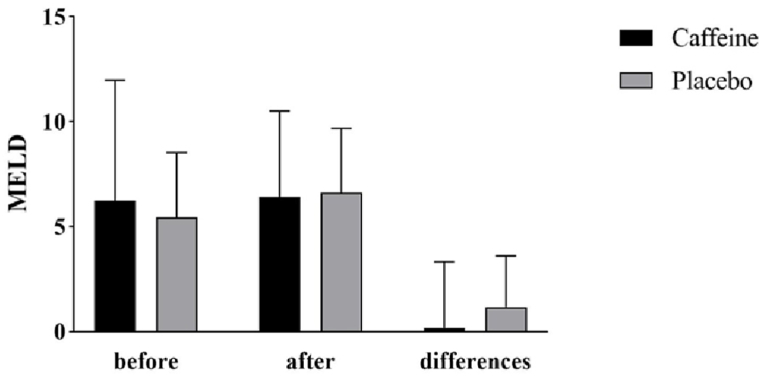
Model for End-stage Liver Disease (MELD) score and its changes in cirrhotic patients in the caffeine group and the placebo group.

As shown in Fig. 2, at the beginning and end of the study, the fibrosis 4 score had no significant difference between the two groups. During the 8 weeks of study, caffeine significantly reduced fibrosis 4 score and placebo significantly increased the fibrosis 4 score. After adjusting the baseline value of the variable, the results indicated a significant decrease in fibrosis 4 score following caffeine supplementation (−0.84 ± 0.89 vs 0.61 ± 1.08, P < 0.001). In Fig. 3, APRI changes are shown. The difference between the two groups at the beginning and the end of the study was not significant. This index showed a significant decrease in the caffeine group and significant increase in the placebo group. A comparison between the two groups indicates that caffeine significantly reduced APRI (−0.25 ± 0.21 vs 0.14 ± 0.28, P < 0.001). Fig. 4 shows MELD changes that had increased in both groups, but this change was only significant in the placebo group (0.17 ± 3.15, P = 0.818 vs 1.17 ± 2.43, P = 0.034).

## 4 Discussion

This is the first randomized, double-blind, placebo-controlled trial of caffeine supplementation performed in patients with cirrhosis. The results of the present study showed that supplementation with 400 mg of caffeine for 8 weeks significantly reduced the inflammatory biomarkers in cirrhotic patients, and a significant improvement in the fibrosis 4 score and APRI was also observed in these patients. These findings indicate that caffeine can be recommended as an adjunct part of other lifestyle modifications in the management of cirrhotic patients.

The association of decaffeinated and regular coffee consumption with cirrhosis has been investigated mainly in observational studies, and most of them reported that coffee has beneficial effects on the liver. However, as far as we know, no clinical trial has attempted to assess the effect of caffeine on inflammatory biomarkers and liver outcomes. Corrao et al. found that higher coffee consumption was associated with a reduced risk of cirrhosis, although the study failed to show this effect for other caffeinated beverages [20]. The relationship between coffee and other beverages, including methyl xanthine, with liver cirrhosis was evaluated in another study in Italy. The results of this study indicated that the risk of cirrhosis in people who consumed one cup of coffee was 33 %, those who consumed two cups of coffee were 43 %, and those who consumed three or more cups were 71 % less than those who did not consume coffee. In this study, no relationship between decaffeinated coffee and liver cirrhosis was detected [23]. Similarly, in a cohort study with a total of 1820201 person-years of observation (125580 individuals with mean follow-up time 14.1 years), the association between coffee consumption and risk of hepatic cirrhosis (199 cases) were evaluated. This study showed that, compared to people who did not consume coffee, the risk of cirrhosis was 30 % lower in people who consumed one cup of coffee per day, 40 % lower in those who consumed one to three cups of coffee, and 80 % lower in those who consumed four cups or more. For nonalcoholic cirrhosis, the risk ratio was 1.2 for one cup or less, 1.3 for one to three cups, and 0.7 for 4 or more cups of coffee [24]. According to observational studies consumption of more than 3 cups of coffee per day has protective effects on the liver. In the present study, the amount of supplemented caffeine was 400 mg/day, which equates to the amount of caffeine found in 4 cups of coffee, which seems to be enough to show positive effects. The protective effects of caffeine on liver function might be due to stimulation of beta oxidative lipolysis, and suppression of oxidative stress [25]. Although most of the studies were consistent with the results of the present study, a recent study failed to show the positive effects of coffee on the liver probably due to low dosage of supplements [26].

In confirmation of the study hypothesis, it was observed that caffeine had a significant effect on inflammatory factors including CRP, IL-6 and TNF-α after 2 months of supplementation. Actually, reactive oxygen species (ROS) stimulate the production of TNF-α, which in turn impairs cell function, resulting in liver fibrosis and necrosis. Caffeine, by increasing the activity of peroxisome proliferator-activated receptor alpha (PPAR-α) and carnitine-palmitoyl transferase 1 (CPT1), on the one hand, reduces lipogenesis and stimulates beta-oxidation of lipids, and on the other hand exhibits anti-inflammatory and antioxidant effects due to the activation of lipoprotein lipase and thus reducing fat accumulation [27]. Contrary to these results, a recent systematic review has reported predominant anti-inflammatory action of coffee but not of caffeine consumption [28]. One of the possible reasons for this contradiction could be in the study participants. The dose of caffeine is another factor.

Furthermore, significant effect was found on liver outcomes in our study which is consistent with the significant effects of caffeine on inflammatory biomarkers reduction. Hepatic stellate cells (HSC) are activated following hepatocyte injury and differentiate into myofibroblast-like cells, leading to liver fibrosis. Caffeine with antifibrotic properties prevents the activation of these cells and liver fibrosis [29]. Since few studies have evaluated the effect of caffeine on liver outcomes and there is a great difference in study design, it is not possible to draw conclusions about the anti-inflammatory and anti-fibrotic effects of caffeine in cirrhotic patients.

The current study has some strengths. It was the first double-blind, randomized, placebo-controlled, clinical trial which has evaluated the effects of caffeine on patients with cirrhosis. Using the caffeine supplement instead of coffee provided this advantage of rolling out the confounding effects of the other components of coffee, its preparation methods, and environmental effects of coffee consumption. Our results might be more accurate because our study population consumed low caffeine. The small sample size and short duration of the study were limitations of this study.

## 5 Conclusion

In conclusion, eight weeks supplementation with 400 mg/day of caffeine had significant impact on markers of hepatic fibrosis, liver functional tests and inflammatory factors in patients with cirrhosis. Moreover, 400 mg/day caffeine was safe so that we have not received any report of side effects from the participants during the study period. Further trials with higher dosages, longer duration, and different active components of coffee are highly recommended.

## CRediT authorship contribution statement

**Seyed Ali Abbas-Hashemi:** Writing – original draft, Investigation, Formal analysis, Data curation, Conceptualization. **Zahra Yari:** Methodology, Investigation, Formal analysis, Data curation. **Behzad Hatami:** Methodology, Investigation. **Amir Anushiravani:** Methodology, Investigation. **Shadi Kolahdoozan:** Project administration, Investigation. **Ali Zamanian:** Investigation. **Nadia Akbarzadeh:** Investigation. **Azita Hekmatdoost:** Writing – review & editing, Supervision, Methodology, Investigation, Funding acquisition, Formal analysis, Data curation, Conceptualization.

## Ethical approval

The ethics committee of the National Nutrition and Food Technology Research Institute approved the study.

## Data availability

Data will be made available by reasonable request to corresponding author.

## Funding

This study is financially supported by 10.13039/501100005851Shahid Beheshti University of Medical Sciences, Tehran, Iran.

## Declaration of competing interest

There is no conflict of interest.

## References

[1] Yoshiji H., Nagoshi S., Akahane T. (2021). Evidence-based clinical practice guidelines for Liver Cirrhosis 2020. J. Gastroenterol..

[2] Stasi C., Silvestri C., Voller F. (2015). Epidemiology of liver cirrhosis. Journal of clinical and experimental hepatology.

[3] Safaei A., Arefi Oskouie A., Mohebbi S.R. (2016). Metabolomic analysis of human cirrhosis, hepatocellular carcinoma, non-alcoholic fatty liver disease and non-alcoholic steatohepatitis diseases. Gastroenterology and hepatology from bed to bench.

[4] Tsochatzis E.A., Bosch J., Burroughs A.K. (2014). Liver cirrhosis. Lancet.

[5] de Freitas Lima L., de Faria Ghetti F., Hermsdorff H. (2020). Dietary total antioxidant capacity is positively associated with muscular strength in cirrhotic outpatients: a cross‐sectional study. J. Hum. Nutr. Diet..

[6] de Oliveira D.G., de Faria Ghetti F., Moreira A.P.B. (2019). Association between dietary total antioxidant capacity and hepatocellular ballooning in nonalcoholic steatohepatitis: a cross-sectional study. Eur. J. Nutr..

[7] Yao C.K., Fung J., Chu N.H.S. (2018). Dietary interventions in liver cirrhosis. J. Clin. Gastroenterol..

[8] Larter C.Z., Yeh M.M., Haigh W.G. (2013). Dietary modification dampens liver inflammation and fibrosis in obesity-related fatty liver disease. Obesity.

[9] Dorvigny B.M., Tavares L.S., de Almeida I.A. (2022). Antiinflammatory and antiinfective effect of caffeine in a mouse model of disseminated salmonellosis. Phytother Res. : PTR.

[10] Svilaas A., Sakhi A.K., Andersen L.F. (2004). Intakes of antioxidants in coffee, wine, and vegetables are correlated with plasma carotenoids in humans. The Journal of nutrition.

[11] Nawrot P., Jordan S., Eastwood J. (2003). Effects of caffeine on human health. Food Addit. Contam..

[12] Heath R.D., Brahmbhatt M., Tahan A.C. (2017). Coffee: the magical bean for liver diseases. World J. Hepatol..

[13] Klatsky A.L., Morton C., Udaltsova N. (2006). Coffee, cirrhosis, and transaminase enzymes. Arch. Intern. Med..

[14] Degertekin B., Tozun N., Soylemez A.G. (2020). Regular coffee intake improves liver enzyme levels and liver histology in patients with chronic alcohol consumption, non-alcoholic fatty liver and non-alcoholic steatohepatitis: report of 259 cases. Hepatology forum.

[15] Bravi F., Bosetti C., Tavani A. (2007). Coffee drinking and hepatocellular carcinoma risk: a meta-analysis. Hepatology.

[16] Bravi F., Bosetti C., Tavani A. (2013). Coffee reduces risk for hepatocellular carcinoma: an updated meta-analysis. Clin. Gastroenterol. Hepatol. : the official clinical practice journal of the American Gastroenterological Association.

[17] Hayat U., Siddiqui A.A., Okut H. (2021). The effect of coffee consumption on the non-alcoholic fatty liver disease and liver fibrosis: a meta-analysis of 11 epidemiological studies. Ann. Hepatol..

[18] Gordillo-Bastidas D., Oceguera-Contreras E., Salazar-Montes A. (2013). Nrf2 and Snail-1 in the prevention of experimental liver fibrosis by caffeine. World J. Gastroenterol..

[19] Anty R., Marjoux S., Iannelli A. (2012). Regular coffee but not espresso drinking is protective against fibrosis in a cohort mainly composed of morbidly obese European women with NAFLD undergoing bariatric surgery. J. Hepatol..

[20] Corrao G., Zambon A., Bagnardi V. (2001). Coffee, caffeine, and the risk of liver cirrhosis. Ann. Epidemiol..

[21] Costentin C.E., Roudot-Thoraval F., Zafrani E.S. (2011). Association of caffeine intake and histological features of chronic hepatitis C. J. Hepatol..

[22] Nouri-Vaskeh M., Malek Mahdavi A., Afshan H. (2020). Effect of curcumin supplementation on disease severity in patients with liver cirrhosis: a randomized controlled trial. Phytother Res. : PTR.

[23] Gallus S., Tavani A., Negri E. (2002). Does coffee protect against liver cirrhosis?. Ann. Epidemiol..

[24] Klatsky A.L., Morton C., Udaltsova N. (2006). Coffee, cirrhosis, and transaminase enzymes. Arch. Intern. Med..

[25] Contaldo F., Santarpia L., Pasanisi F. (2019). Chronic inflammatory liver diseases and coffee intake. Curr. Opin. Clin. Nutr. Metab. Care.

[26] Mansour A., Mohajeri-Tehrani M.R., Samadi M. (2021). Effects of supplementation with main coffee components including caffeine and/or chlorogenic acid on hepatic, metabolic, and inflammatory indices in patients with non-alcoholic fatty liver disease and type 2 diabetes: a randomized, double-blind, placebo-controlled, clinical trial. Nutr. J..

[27] Montagner A., Polizzi A., Fouché E. (2016). Liver PPARα is crucial for whole-body fatty acid homeostasis and is protective against NAFLD. Gut.

[28] Paiva C., Beserra B., Reis C. (2019). Consumption of coffee or caffeine and serum concentration of inflammatory markers: a systematic review. Crit. Rev. Food Sci. Nutr..

[29] Shim S.G., Jun D.W., Kim E.K. (2013). Caffeine attenuates liver fibrosis via defective adhesion of hepatic stellate cells in cirrhotic model. J. Gastroenterol. Hepatol..

